# Potential Novel Thioether-Amide or Guanidine-Linker Class of SARS-CoV-2 Virus RNA-Dependent RNA Polymerase Inhibitors Identified by High-Throughput Virtual Screening Coupled to Free-Energy Calculations

**DOI:** 10.3390/ijms222011143

**Published:** 2021-10-15

**Authors:** Marko Jukič, Dušanka Janežič, Urban Bren

**Affiliations:** 1 Laboratory of Physical Chemistry and Chemical Thermodynamics, Faculty of Chemistry and Chemical Engineering, University of Maribor, Smetanova 17, SI-2000 Maribor, Slovenia; marko.jukic@um.si; 2 Faculty of Mathematics, Natural Sciences and Information Technologies, University of Primorska, Glagoljaška 8, SI-6000 Koper, Slovenia

**Keywords:** COVID-19, SARS-CoV-2 virus, RNA-dependent RNA polymerase, binding site identification, virtual screening, non-covalent inhibitors, in silico drug design, molecular dynamics, free-energy calculations, ensemble docking

## Abstract

SARS-CoV-2, or severe acute respiratory syndrome coronavirus 2, represents a new pathogen from the family of *Coronaviridae* that caused a global pandemic of COVID-19 disease. In the absence of effective antiviral drugs, research of novel therapeutic targets such as SARS-CoV-2 RNA-dependent RNA polymerase (RdRp) becomes essential. This viral protein is without a human counterpart and thus represents a unique prospective drug target. However, in vitro biological evaluation testing on RdRp remains difficult and is not widely available. Therefore, we prepared a database of commercial small-molecule compounds and performed an in silico high-throughput virtual screening on the active site of the SARS-CoV-2 RdRp using ensemble docking. We identified a novel thioether-amide or guanidine-linker class of potential RdRp inhibitors and calculated favorable binding free energies of representative hits by molecular dynamics simulations coupled with Linear Interaction Energy calculations. This innovative procedure maximized the respective phase-space sampling and yielded non-covalent inhibitors representing small optimizable molecules that are synthetically readily accessible, commercially available as well as suitable for further biological evaluation and mode of action studies.

## 1. Introduction

In late 2019–early 2020, scientific and medicinal communities identified a novel human pathogen—severe acute respiratory syndrome coronavirus 2 (SARS-CoV-2) causing a pandemic named COVID-19 [1]. This pathogen belongs to the *Coronaviridae* family and represents a positive-sense single-stranded RNA (+ssRNA) virus [2,3]. Previous occurrences of related pathogens can be traced to 2003 when coronaviruses were reported to cause severe acute (SARS) and Middle East (MERS) respiratory syndromes [4,5,6]. The COVID-19 disease is of grave global concern because, while the majority of cases display mild symptoms, a variable percentage (0.2 to >5%) of patients progress to pneumonia and multi-organ failure leading to potential death, especially without medical assistance [7,8,9,10,11,12].

At the time, there were no registered drugs against SARS-CoV-2, and only several drug repurposing strategies have been reported [13,14,15,16,17,18,19,20,21,22,23,24]. Current therapeutic options include direct-acting remdesivir, symptomatic treatment or immunomodulatory approaches mainly targeting host responses (dexamethasone, tocilizumab, sarilumab mitigating IL-6 response) [25,26,27,28]. Many clinical studies are ongoing, albeit also with disadvantageous results such as the recent retractions of chloroquine and hydroxychloroquine drugs suggested for the treatment of COVID-19 [29].

In the context of only a handful of therapeutic options to fight this global threat, we performed a novel drug design and virtual screening study in order to identify potential inhibitors of the RNA-dependent RNA polymerase (RdRp) of SARS-CoV-2. RdRp catalyzes the synthesis of an RNA strand complementary to a given RNA template [30,31]. This essential viral enzyme is highly conserved among (+ssRNA) viral species and, as such, devoid of a human counterpart promoting this protein to a viable, under-investigated and prospective therapeutic target [32,33]. Moreover, the complexity of RNA replication machinery involving a synchronized action of multiple viral and host proteins towards final RNA polymerization makes this target a difficult system for designing in vitro biological experiments [34,35]. Furthermore, current postulated actives on RdRp rely on (infected) whole cell-line experimental data and on conducted preclinical in vivo data, further emphasizing the need for a thorough in silico computer-assisted drug design approach [36,37].

**Figure 1:**
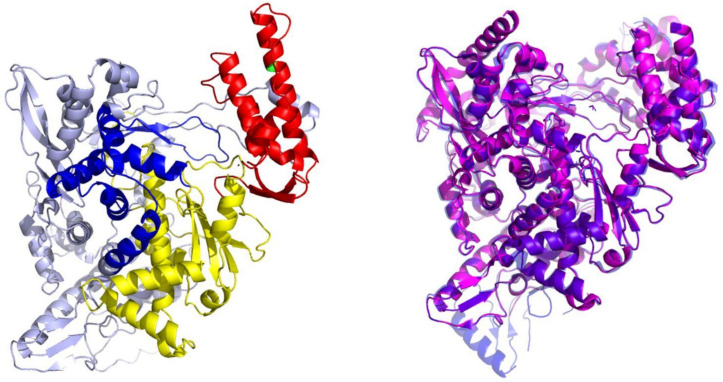
(**Left**) Structure of a typical viral RdRp. The protein is presented in a cartoon model with thumb subdomain colored in red, palm subdomain in yellow and fingers subdomain in blue. The RNA channel groove is located in the central cavity encircled by all three subdomains. (**Right**) Superimposition of the SARS-CoV (PDB ID: 6NUR, magenta colored cartoon model) and the recently published cryoEM of SARS-CoV-2 RdRp (PDB ID: 6M71, blue colored cartoon model) revealing a virtually identical structural architecture.

The RdRp forms a part of the Replicase polyprotein 1ab (Uniprot P0DTD1, sequence location 4393-5324; nsp12). Based on the cryoEM structure of the SARS-CoV reported by Kirchdorfer et al., RdRp contains a polymerase domain described in the literature as “right hand” (PDB ID: 6NUR numbering; 398–919) similar to other polymerases [38]. The domain itself consists of fingers (398–581, 628–687), palm (582–627, 688–815) and thumb (816–919) subdomains (Figure 1). The palm subdomain, composed of a four-stranded antiparallel beta sheet and two alpha helices, represents the most conserved structural motif. The SARS-CoV nsp12 also contains two structural Zn binding sites, both distal to the active site and not involved in any protein–protein or protein–RNA interactions. While the outer surface of the protein is predominantly negatively charged, the RNA binding grove carries a net positive charge. The active site of RdRps is in general highly conserved and includes two successive aspartate residues protruding from a beta-turn at the nucleotide channel of the palm subdomain [39,40].

The SARS-CoV (PDB ID: 6NUR, nsp12, chain A) and SARS-CoV-2 (P0DTD1, RdRp) RdRp amino-acid sequences are 94% similar as calculated by the Clustal software (identical positions 897, similar positions 30, identity: 93.9%) [41]. Moreover, examining the recently reported cryoEM structures of SARS-CoV-2 RdRp by Gao et al. (PDB ID: 6M71 and 7B3D), the structural conservation to SARS-CoV RdRp (all atom RMSD of 0.6 Å) can be clearly observed as well (Figure 1) [42,43]. The binding mode of RdRp is described by Yin et al. (PDB ID: 7BV2) [44].

**Figure 2:**
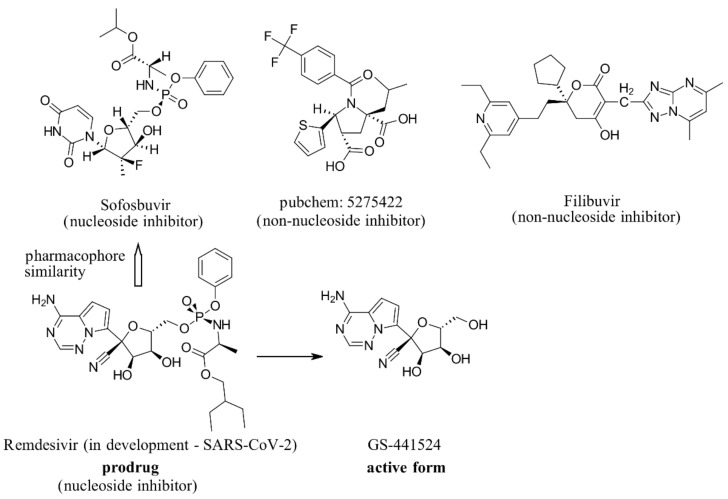
Some of the existing nucleoside and non-nucleoside inhibitors of RdRps found in the scientific literature primarily developed against HCV.

RdRp has been already targeted in different RNA viruses, including Hepatitis C Virus (HCV), Norovirus, Zika Virus (ZIKV), and coronaviruses (CoVs) [45,46,47,48]. Nevertheless, only a single drug, sofosbuvir, is registered with HCV indication (RdRp pharmacodynamics), and a handful of nucleoside and non-nucleoside inhibitors are under investigation as reviewed by Powrill et al. (Figure 2) [49]. The binding modes of developed inhibitors are elaborated in the literature [50,51]. Moreover, remdesivir, a prodrug currently authorized for emergency use for the SARS-CoV-2 treatment, shares its pharmacophoric traits with the registered drug sofosbuvir and represents a prime example of a viral nucleoside RdRp inhibitor [52,53,54].

Viability of RdRp as a drug target has been extensively studied beforehand in silico by Aftab et al. [55]. We firmly believe the SARS-CoV-2 RdRp represents a validated and underexplored target, supported by experimental data for the development of potential anti COVID-19 drugs, especially in the field of non-covalent, non-nucleoside RNA-dependent RNA polymerase small-molecule inhibitors (Figure 2).

## 2. Database Preparation

**Figure 3:**
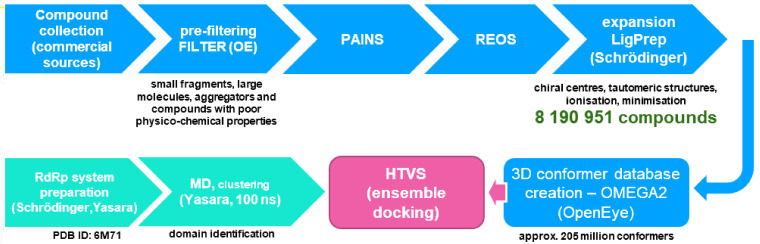
Database preparation for the subsequent high-throughput virtual screening (HTVS) on the SARS-CoV-2 RNA-dependent RNA polymerase (RdRp, PDB ID: 6M71). The final database before conformer generation contained 8 190 951 molecules.

We started preparing an in-house contemporary in silico database suitable for VS (virtual screening) or HTVS (high-throughput virtual screening) applications. This database is by its design tailored towards drug-like, small-molecule selection of synthetically accessible and commercially available compounds that should also facilitate fast analogue collection or preparation as well as efficient CPU-time in downstream calculations (Figure 3).

We combined various commercial compound sources (e.g., ENAMINE, Vitas-M, Chembridge, Maybridge, Ambinter, Otava, PrincetonBIO, Key-Organics, Life Chemicals, Uorsy, Specs) and pre-filtered all compounds in order to exclude small fragments or extra-large molecules, aggregators, and compounds with poor physico-chemical properties. This step was performed using OpenEye FILTER software (OpenEye Scientific Software, Inc., Santa Fe, NM, USA) and the following parameters: min_molwt 250, max_molwt 800, min_solubility moderately, eliminate known and predicted aggregators and allowed elements: H, C, N, O, F, S, Cl, Br, I, P. The obtained database was subsequently filtered for *pains* (pan-assay interference compounds) [56,57] and *reos* (rapid elimination of swill) structures in order to eliminate reactive and labile functional groups. Therefore, Knime software with RDKit software nodes was applied to compare all structures in the library to the selection of SMARTS-formatted flags and to remove hits from the database [58].

We ended up with a collection of approximately 4 million compounds that were expanded in the subsequent step where final enumeration of undefined chiral centers, tautomeric structures, removal of structural faults, ionization at the pH of 7.4 and minimization (using OPLS3 force-field) towards the final 3D conformation was performed [59]. For this step, the Ligprep tool by Schrödinger (Release 2021-1, Schrödinger, LLC, New York, NY, USA, 2021) was employed [60,61]. The final database thus consisted of 8 190 951 molecules and was ultimately used for conformer 3D-database preparation with the OpenEye OMEGA2 tool (OpenEye Scientific Software, Inc., Santa Fe, NM, USA). The maximum number of conformations was set at 25 and RMSD threshold of 0.8 Å afforded 205 million compound conformations ready for HTVS.

## 3. Molecular Dynamics and Clustering

**Figure 4:**
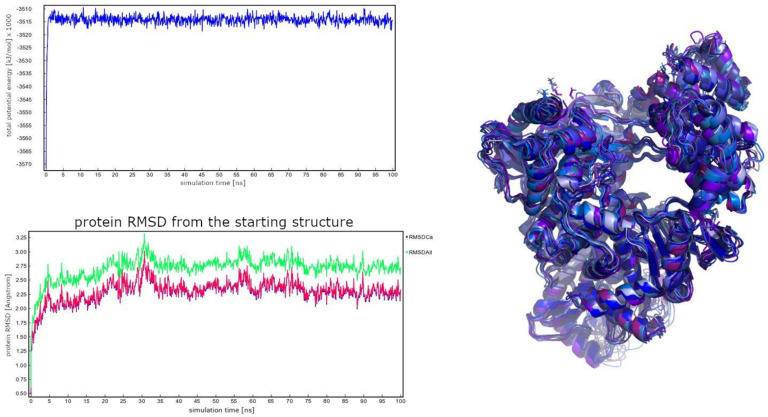
(**Left**) total potential energy of the system and protein RMSD during the 100 ns MD trajectory (red line depicts CA atoms and green line all atoms). (**Right**) Superimposition of cluster centroids obtained using the ClusCo software representing the observed conformational flexibility of the SARS-CoV-2 RdRp.

The available Cryo-electron microscopy SARS-CoV-2 RNA-dependent RNA polymerase in complex (four protein chains, 1122 residues, 17800 atoms) with cofactors (PDB ID: 6M71; Resolution: 2.90 Å; by Gao et al. [42,62]) was prepared with Yasara (19.6.6.L.64) software [63]. Missing hydrogens were added, overlapping atoms adjusted, missing residues and atoms incorporated, hydrogen bonds optimized, proteins capped (Ace–4 positions, Nma–4 positions) and residue ionization assigned at pH = 7.4 [64,65]. A cubic box (10 Å away from all atoms) was solvated using TIP3P water model (81749 water molecules) and 0.9% of NaCl (234 Na^+^ and 226 Cl^−^ ions) to achieve physiological ionic power and electroneutrality. After removing bumps/clashes with a steepest descent minimization, an annealing minimization was used to reach a stable local energy minimum. From there, the molecular dynamics (MD) simulation was started by assigning random initial velocities and slowly heating the system up to 298 K. A 100 ns simulation using an AMBER14 force field for the solute, GAFF and AM1BCC charges for the ligands as well as TIP3P water was initiated (total number of atoms: 263,547). The equations of motion were integrated with a multiple timestep of 1.25 fs for bonded interactions and 2.5 fs for non-bonded interactions at a temperature of 298 K and a pressure of 1 atm (NPT ensemble, Berendsen baro- and thermostat, coupling to the time average temperature and density) with snapshots saved every 100 ps [63,66]. Long-range electrostatic interactions were calculated with the Particle Mesh Ewald algorithm [67]. The SHAKE algorithm was not applied in the simulation. Energy parameters of the system were stable throughout the production run as was the root-mean-square deviation (RMSD) of the protein backbone. No major conformation changes and chain movements were observed during the production run. MD snapshots in 100 ps intervals were collected, and the 1000 obtained protein conformations were aligned onto the starting snapshot followed by the structural clustering using ClusCo software [68]. Its parameters used were hierarchical clustering in a pairwise average-linkage manner with a backbone RMSD score (Figure 4). Twenty clusters were obtained by the ClusCo, and their centroid structures were considered as protein conformations that represent the flexibility of the SARS-Cov-2 RdRp and were used for ensemble docking in the subsequent step (Figure 4, right).

## 4. Target Preparation

**Figure 5:**
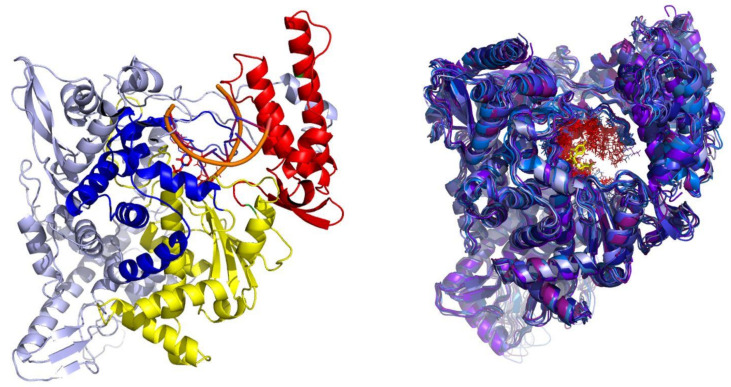
(**Left**) SARS-CoV-2 RdRp identified subdomains. Protein is presented in a cartoon model with the thumb subdomain in red, the palm subdomain in yellow and the fingers subdomain in blue. The RNA channel groove can be found in the central cavity between all three described subdomains (RNA in orange is superimposed from PDB ID: 4WTG for demonstration). (**Right**) Structural superimposition of the clustered centroids representing the conformational flexibility of the SARS-CoV-2 RdRp during the MD experiment. Centrally colored in red is the identified binding site with superimposed sofosbuvir from PDB ID: 4WTG in yellow.

Initial structure alignment and domain identification was performed using NCBI NC_045512 sequence and InterPro, PsiPred and CATH servers [69,70,71]. After structure preparation, we used superimposition of HCV RdRp in complex with sofosbuvir (2′-deoxy-2′-fluoro-2′-methyluridine-5′-(trihydrogen diphosphate) small molecule RdRp inhibitor) published by Appleby, TC et al. (PDB ID: 4WTG) performed by Open Source PyMOL, release 2.1 and Schrödinger Small-Molecule Discovery Suite (Schrödinger LLC, New York, NY, USA) [72]. Sofosbuvir was positioned analogously to the remdesivir postulated by Gao et al. (Figure 5) [42]. The binding site was thus located above the palm and between the finger/thumb subdomains around our reference small-molecule ligand sofosbuvir as described below (Figure 5) [73].

The receptor structure was generated using the MakeReceptor GUI of OEDocking 3.2.0.2 software package (OpenEye Scientific Software, Inc., Santa Fe, NM, USA). A rectangular box with the volume of 9150 Å^3^ (25.00 Å × 18.33 Å × 20.33 Å) was defined around the sofosbuvir ligand superimposed in the previous step. A balanced site shape potential was calculated with docking volume of 1626 Å^3^ without applied central inner volume. No constraints were used, and active site residue conformations as well as protonation states were maintained.

## 5. Virtual Screening

The prepared 3D conformer library of 205 million structures was applied for HTVS with molecular docking into the pre-prepared RdRp receptor using FRED software from OpenEye (OpenEye Scientific Software, Inc., Santa Fe, NM, USA). In this protocol we employed normal-resolution docking parameters and Chemgauss4 scoring function [74,75,76]. HTVS was performed on an Intel^®^ (Santa Clara, CA, USA) Xeon^®^ E5-2630 v4 CPUs and 32 GB of memory machines using the Linux operating system. Statistical analysis of random compound samples (1000 samples taken from the pool of approximately 8 million docked compounds) revealed the docking scores reached a minimum of −13.234 and a maximum of 3.05 with a mean of −5.605 and a standard deviation of 1.233. Normality was tested with a Shapiro–Wilk test using significance level of 0.05. H_0_ was not rejected (*p* value = 0.0536), and normality of FRED scoring values was assumed. Z-scores were therefore calculated for the whole docked library, and −5σ of top scoring compounds were selected for the final ensemble docking (3148 top-scoring compounds; docking score cut-off of −11.77) [77].

The RdRp docking ensemble was created from the clustered set of 20 protein conformations (obtained with ClusCo as already described before). Protein cluster alignment was checked with Theseus software that performed identically and confirmed the suitability of the clustering alignment [78]. Upon examination of each cluster size, 13 protein conformations were selected for ensemble docking as they occupied more than 1% of the total trajectory time (cluster number–portion of the total trajectory time that the conformations of the relevant cluster occupy): 1–0.02, 2–0.04, 3–0.17, 4–0.02, 5–0.06, 6–0.12, 7–0.05, 8–0.19, 9–0.14, 10–0.08, 11–0.05, 12–0.03, 13–0.02. The following clusters were deemed not sufficiently influential for the ensemble docking as they occupied only a negligible portion of the total trajectory time: 14–0.006, 15–0.005, 16–0.004, 17–0.003, 18–0.001, 19–0.001, 20–0.001. Upon model selection, receptors were generated as already described before using MakeReceptor GUI of OEDocking 3.2.0.2 software package (OpenEye Scientific Software, Inc., Santa Fe, NM, USA) with default parameters (the box was resized to 21 Å in the x plane along the cavity surrounded by the palm and the finger subdomains). No constraints were used, and active site residue conformations as well as protonation states were maintained. The −5σ of top scoring compounds were finally re-docked to the 13 representative RdRp conformations using FRED from OpenEye software, and the final ensemble score was calculated by Equation (1).

(1) $$S_{ensamble}=\sum_{N=1}^{13}S_{i}\cdot W_{i}$$

Equation (1): Ensemble docking score calculation where *S_i_* represents individual FRED docking scores and *W_i_* the corresponding ensemble weights, respectively. *W_i_* possesses the following values from 1 to 13 individuals: 0.02, 0.04, 0.05, 0.17, 0.03, 0.02, 0.08, 0.06, 0.12, 0.05, 0.19, 0.02 and 0.14 as identified from the relative cluster probabilities (sizes).

The identified top 200 scoring hits were subjected to clustering analysis in order to obtain representative top-scoring scaffolds. MACCS fingerprints were calculated for all compounds and, after similarity matrix calculation, were binned into 20 representative clusters. The five top-scoring and clustered compounds are presented below in Table 1 (extended hitlist can be found in Supplementary Materials).

## 6. Free-Energy Calculations

From the top five scoring hits, compounds **1**, **2**, **3** and **5** constitute a similar structural class with a central guanidine linker, flanked by two aryl systems, while compound **4** displays a unique central thioether–amide linkage between terminal triazole and pyrrole heterocycles. All compounds also exhibit an analogous calculated binding mode as elaborated in Section 7. Therefore, compound **1** from the first structural class and compound **4** with FRED ensemble docking scores of −12.72 and −11.59, respectively, were selected for subsequent binding free energy calculations. Consequently, the Linear Interaction Energy (LIE) methodology developed by Aqvist et al. was employed [79]. It is especially suitable for ligand–receptor complex interaction studies and reported as superior to the MM/GBSA method commonly employed for this task [80]. To this end, we separately calculated the average VdW and electrostatic interaction energies of ligands **1** and **4** with water (explicit TIP3P solvent model) and of the ligands in complex with the solvated RdRp. Then, binding free energies of ligands **1** and **4** to RdRp were computed using Equation (2).

(2) $$\Delta G_{LIE}^{bind}=\alpha \;\sum_{i}^{N}\Delta V_{i}^{vdW}+\beta \;\overset{N}{\underset{i}{\sum }}\Delta V_{i}^{coulomb}$$

Equation (2): The ∆ term indicates the difference in average interaction energy between the ligand bound (in solvated RdRp) and ligand free (in water) states. The *α* and *β* represent LIE empirical parameters, determined by comparing calculated and experimentally measured binding affinities. Their values were optimized by Aqvist et al. [49].

For the LIE approach the MD simulations of 100 ns of clustered complexes from the ensemble docking (13 complexes for two ligands, 26 in total) as well as of free ligands in water were carried out to obtain the vdW and electrostatic interaction energies between the ligands and their surroundings. Each MD simulation afforded 1000 snapshots (using 100 ps intervals) of each compound in protein bound and water environments for electrostatic and vdW interaction energy calculations. Interaction energies were averaged for the ligand free simulations (two sets separately for compounds **1** and **4**), while ligand bound potential energies were weighted according to Equation (3) and subsequently used in the LIE calculation [81,82,83].

(3) $$W_{i}=\frac{e^{-\Delta G_{calc,\;i}\;/\;k_{b}T}}{\sum_{i}e^{-\Delta G_{calc,\;i}\;/\;k_{b}T}}$$

Equation (3): Weighting of ligand interaction energies as per Aqvist et al. (*k_b_*-Boltzmann constant in J·K^−1^).

Weighted LIE calculation (Table 2) resulted in the favorable affinity towards RdRp for compound **1** with ${\Delta G}_{LIE}^{bind}$ value of −2.6 ± 0.6 and for compound **4** with ${\Delta G}_{LIE}^{bind}$ value of −6.5 ± 0.8 kcal/mol using the preoptimized α and β parameters. Moreover, ligand–protein contact and RMSD analyses along all 13 MD production runs of compounds **1** and **4** confirmed a stable binding conformation and an analogous binding mode.

## 7. Results and Discussion

After the SARS-CoV-2 RNA-dependent RNA polymerase (RdRp) active site identification based on subdomain locations, we postulated that the active site gorge is accessible and free to bind the nucleotides passing through the nucleotide channel. This hypothesis is corroborated by the cryoEM RdRp experimental structure (PDB ID: 6M71) by Gao et al. [42]. Moreover, the active site aspartate residues protruding from a *beta*-turn of the palm subdomain were identified at the RNA channel (PDB ID: 6M71 numbering: Asp760, Asp761), and the superimposition with HCV RdRp (PDB ID: 4WTG) placed its small-molecule inhibitor sofosbuvir (reference) at the active site analogously to the remdesivir binding pose postulated by Gao et al. [42].

**Figure 6:**
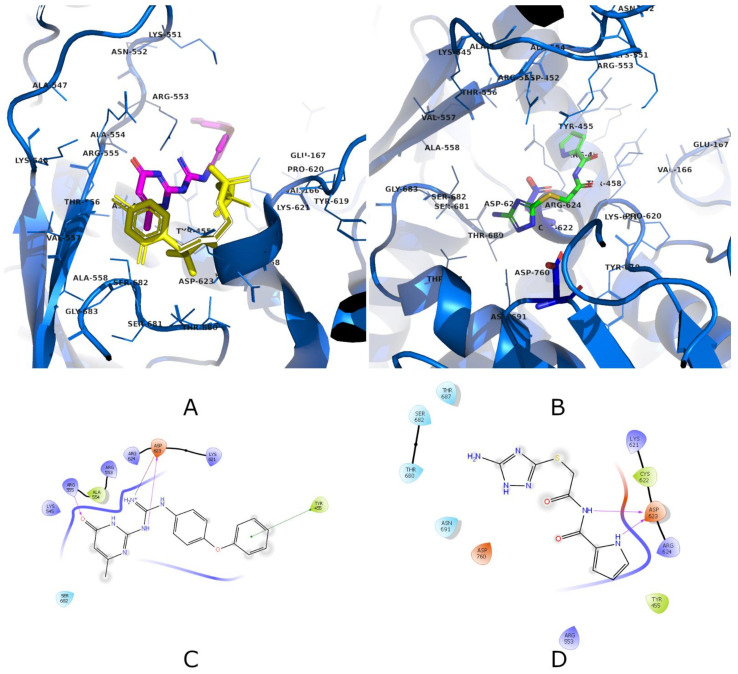
Panel (**A**): The predicted binding mode of Compound **1** (magenta stick model) to the SARS-CoV-2 RdRp (sofosbuvir reference ligand colored in yellow); Panel (**B**): The predicted binding mode of Compound **4** (green stick model) in the vicinity of the key Asp623 and Asp760 residues; Panel (**C**): Compound **1** in the SARS-CoV-2 RdRp active site in a 2D projection; Panel (**D**): Compound **4** in the SARS-CoV-2 RdRp active site in a 2D projection.

All docked compounds were positioned at the bottom of the nucleotide binding gorge sandwiched between the key Asp623 and Asp760 residues. All top-scoring compounds displayed a similar binding motif; therefore, the best-scoring compound **1** from Table 1 will be discussed in more detail. Compound **1** forms hydrogen bonds of its central guanidine linker with key Asp623, Lys621 and Arg553 as well as hydrophobic contacts with Arg553 and Lys621 (Figure 6). The central linker also forms a salt bridge with Asp623, while the compound **1** pyrimidine-4-one moiety is interacting with Arg555, but analogous binding modes of the remaining top-scoring compounds extend the terminal part of their molecules towards the Lys621 and Tyr455 pocket as well. All compounds make use of described distal pockets with their cyclic and/or aromatic moieties while placing their central linker or terminal moieties in the vicinity of the key Asp632 and Asp760 residues. Compounds also display a propensity to establish π–π interactions with Tyr455 or cat-π interactions with Lys621. An elegant direct ensemble docking–Linear Interaction Energy coupling methodology was employed to assess the binding propensity of the identified hit compounds as described beforehand [84]. All presented compounds are stably positioned near the postulated *remdesivir* binding pose, exhibit favorable ${\Delta G}_{LIE}^{bind}$ values and can effectively block the RNA matrix access to the RdRp catalytic pocket when bound in the predicted conformation.

## 8. Conclusions

This paper reports a successful leverage of known structural data on related viral RdRp enzymes to postulate the SARS-CoV-2 RdRp active site position. Next, a HTVS in silico campaign coupled to free-energy calculations was commenced. The innovative coupled procedure maximized the respective phase-space sampling and identified potential small-molecule, non-covalent, synthetically accessible and commercially available RdRp inhibitors. The top-scoring compounds possess two terminal aromatic moieties (e.g., pyrimidine, triazol or benzene analogues on one side in conjunction with benzene, pyrrole or indole on the other side) merged by a central nitrogen-rich guanidine or thioether–amide linker. Compounds display favorable binding free energies and uniform binding motifs at the RdRp nucleotide gorge with their central linker sandwiched between the key Asp623 and Asp760 amino-acid residues, effectively forming a novel class of RdRp inhibitors. We firmly believe that this work will facilitate further research into SARS-CoV-2 RdRp inhibitor design and provide the groundwork for the biological evaluation of innovative non-nucleoside RdRp inhibitors with the ultimate goal of effectively developing new probes for SARS-CoV-2 studies or novel drugs against COVID-19 and related viral diseases.

## Figures and Tables

**Table 1:** The top five hits from the high-throughput virtual screening (HTVS) on the SARS-CoV-2 RdRp together with three nucleoside mimic RdRp inhibitors for comparison. The extended list is provided in Supplementary Materials.

No.	Structure	Mr (g/mol)	Smiles	Cluster	Ensemble Docking Score
1	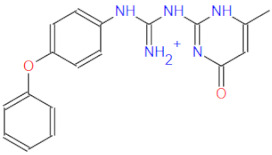	336.4	CC(NC(NC(Nc(cc1)ccc1Oc1ccccc1)=[NH2+])=N1)=CC1=O	13	−12.72
2	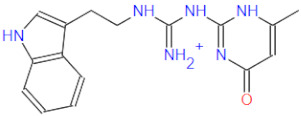	311.4	CC(N=C(NC(NCCc1c[nH]c2c1cccc2)=[NH2+])N1)=CC1=O	11	−12.68
3	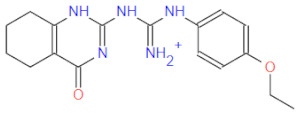	328.4	CCOc(cc1)ccc1NC(NC(NC1=C2CCCC1)=NC2=O)=[NH2+]	15	−12.23
4	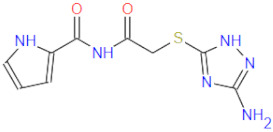	266.3	Nc1n[nH]c(SCC(NC(c2ccc[nH]2)=O)=O)n1	2	−11.59
5	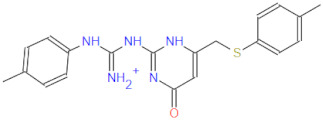	380.5	Cc(cc1)ccc1NC(NC(NC(CSc1ccc(C)cc1)=C1)=NC1=O)=[NH2+]	14	−11.51
6 *	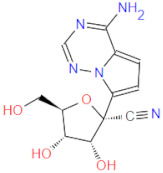 GS-441524 (remdesivir metabolite)	291.3	C1(C(N)=N2)N(N=C2)C(=CC=1)[C@@](O1)(C#N)[C@@H]([C@H](O)[C@H]1CO)O	/	−10.96
7 *	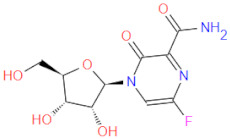 favipiravir-ribose	289.2	[C@@H]1(N(C=C2F)C(=O)C(=N2)C(=O)N)O[C@H](CO)([C@@H](O)[C@H]1O)	/	−10.78
8 *	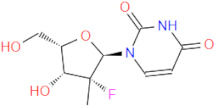 GS-461203 (sofosbuvir metabolite)	260.2	C1=CC(NC(=O)N1[C@H](O[C@H]1CO)[C@](C)(F)[C@@H]1O)=O	/	−10.24

* compounds docked as phosphates and depicted herein for comparison.

**Table 2:** Calculation of binding free energies for compounds **1** and **4** using the LIE methodology.

Compound	Free VdW (Kcal/Mol)	Free Coulomb (Kcal/Mol)	Complex VdW Weighted Sum (Kcal/Mol)	Complex Coulomb Weighted Sum (Kcal/Mol)	$\Delta G_{LIE}^{BIND}\;(\mathrm{Kcal}/\mathrm{Mol})$
**1**	−6.0 ± 0.1	−19.8 ± 0.2	−9.3 ± 0.5	−19.1 ± 0.6	−2.6 ± 0.6
**4**	−3.4 ± 0.1	−46.0 ± 0.8	−6.6 ± 0.1	−49.4 ± 0.8	−6.5 ± 0.8

## Data Availability

The extended hitlist of compounds is available in the Supplementary Materials.

## Supplementary Materials

The following are available online at https://www.mdpi.com/article/10.3390/ijms222011143/s1.

## Abbreviations

MD: molecular dynamics; RdRp: RNA-dependent RNA polymerase; VS: virtual screening; HTVS: high throughput virtual screening; HCV: Hepatitis C Virus; RMSD: root mean square deviation.
