# Supplementary material for: Potential Novel Thioether-Amide or Guanidine-Linker Class of SARS-CoV-2 Virus RNA-Dependent RNA Polymerase Inhibitors Identified by High-Throughput Virtual Screening Coupled to Free-Energy Calculations
Source: Int J Mol Sci. 2021 Oct 15;22(20):11143. doi: 10.3390/ijms222011143 (PMC8540652; doi:10.3390/ijms222011143)
Supplement: Supplementary file 1 [file ijms-22-11143-s001.zip › ijms-1409846-supplementary.pdf]

Supporting Information

for

Potential Novel Thioether-Amide or Guanidine-Linker Class of SARS-CoV-2 Virus RNA-Dependent RNA Polymerase Inhibitors Identified by High-Throughput Virtual Screening Coupled to Free-Energy Calculations

Marko Jukič [a, b], Dušanka Janežič [b], and Urban Bren [a,b*]*

[a] University of Maribor, Faculty of Chemistry and Chemical Engineering, Laboratory of Physical Chemistry and Chemical Thermodynamics, Smetanova 17, SI-2000 Maribor, Slovenia.

[b] University of Primorska, Faculty of Mathematics, Natural Sciences and Information Technologies, Glagoljaška 8, SI-6000 Koper, Slovenia.

Authors to whom correspondence should be addressed:

* Urban Bren, University of Maribor, Faculty of Chemistry and Chemical Engineering, Laboratory of Physical Chemistry and Chemical Thermodynamics, Smetanova 17, SI-2000 Maribor, Slovenia; E-mail: urban.bren@um.si.

* Dušanka Janežič, University of Primorska, Faculty of Mathematics, Natural Sciences and Information Technologies, Glagoljaška 8, SI-6000 Koper, Slovenia; E-mail: dusanka.janezic@upr.si.

Table S1: Extended list of hit compounds.

no.	name	SMILES	AMW (g/mol)	docking score
1	omega_95307	Cc1cc(=O)nc(NC(=[NH2+])Nc2ccc(Oc3ccccc3)cc2)[nH]1	336.375	-12.724622260000002
2	omega_61132	Cc1cc(=O)[nH]c(NC(=[NH2+])NCCc2c[nH]c3ccccc23)n1	311.369	-12.680652000000002
3	omega_95597	CCOc1ccc(NC(=[NH2+])Nc2nc(=O)c3c([nH]2)CCCC3)cc1	328.39600000000013	-12.230796840000002
4	omega_23267	Nc1n[nH]c(SCC(=O)NC(=O)c2ccc[nH]2)n1	266.286	-11.58801522
5	omega_266712	Cc1ccc(NC(=[NH2+])Nc2nc(=O)cc(CSc3ccc(C)cc3)[nH]2)cc1	380.49700000000007	-11.50689556
6	omega_79908	CC(C)Oc1ccc(CNC(=[NH2+])NC(N)=[NH2+])cc1Cl	285.779	-11.45967399
7	omega_212413	Cc1ccc(NC(=[NH2+])NC2=[NH+][C@H](CC(=O)Nc3ccccc3C)C(=O)N2)cc1	380.4520000000001	-11.412305480000002
8	omega_62575	CCOC(=O)CCc1c(C)nc(NC(=[NH2+])Nc2ccccc2)[nH]c1=O	344.39500000000004	-11.39842637
9	omega_200646	[NH3+][C@H](CCc1nc2ccccc2[nH]1)c1nc2ccccc2[nH]1	292.36600000000004	-11.39447159

10	omega_196053	C[C@@H](c1nc2ccccc2c(=O)[nH]1)[N@@H+](C)Cc1nc(=O)c2ccccc2[nH]1	362.413	-11.346182480000001
11	omega_62577	CCOC(=O)CCc1c(C)nc(NC(=[NH2+])Nc2ccc(C)c(C)c2)[nH]c1=O	372.4490000000001	-11.33483645
12	omega_245800	CCCCOc1ccc(CNC(=[NH2+])NC(N)=[NH2+])cc1	265.361	-11.28781721
13	omega_97605	NC(=[NH2+])NCCC[C@@H](NC(=O)c1ccccc1)C(=O)[O-]	278.31200000000007	-11.278012140000001
14	omega_95279	NC(=[NH2+])NC(=[NH2+])Nc1ccc(Br)cc1	258.123	-11.275575360000001
15	omega_187544	NC(=[NH2+])NC(=[NH2+])Nc1ccc(Br)cc1	258.123	-11.275575360000001
16	omega_103218	Cc1cc(Br)ccc1NC(=[NH2+])NC(N)=[NH2+]	272.1500000000001	-11.253177930000003
17	omega_226153	NC(=[NH2+])NCCC[C@H](NC(=O)c1ccccc1)C(N)=O	278.33600000000007	-11.23412681
18	omega_286631	CCOc1ccc(NC(=[NH2+])Nc2nc(O)c3c(n2)CCC3)cc1	314.3690000000001	-11.17112752

19	omega_213103	[NH2+]=C(NC1=[NH+][C@H](CC(=O)Nc2cc(Cl)cc(Cl)c2)C(=O)N1)Nc1ccccc1	421.28800000000007	-11.142613710000003
20	omega_293671	COc1ccc(NC(=[NH2+])Nc2nc(C)c(CC#N)c(O)n2)cc1	313.341	-11.138971360000001
